# Parasitic Protozoa and Interactions with the Host Intestinal Microbiota

**DOI:** 10.1128/IAI.00101-17

**Published:** 2017-07-19

**Authors:** Stacey L. Burgess, Carol A. Gilchrist, Tucker C. Lynn, William A. Petri

**Affiliations:** Division of Infectious Diseases and International Health, Department of Medicine, University of Virginia, Charlottesville, Virginia, USA; University of Florida

**Keywords:** microbiota, parasite, host pathogen, protozoa

## Abstract

Parasitic protozoan infections represent a major health burden in the developing world and contribute significantly to morbidity and mortality. These infections are often associated with considerable variability in clinical presentation. An emerging body of work suggests that the intestinal microbiota may help to explain some of these differences in disease expression. The objective of this minireview is to synthesize recent progress in this rapidly advancing field. Studies of humans and animals and *in vitro* studies of the contribution of the intestinal microbiota to infectious disease are discussed. We hope to provide an understanding of the human-protozoal pathogen-microbiome interaction and to speculate on how that might be leveraged for treatment.

## INTRODUCTION

**FIG 1:**
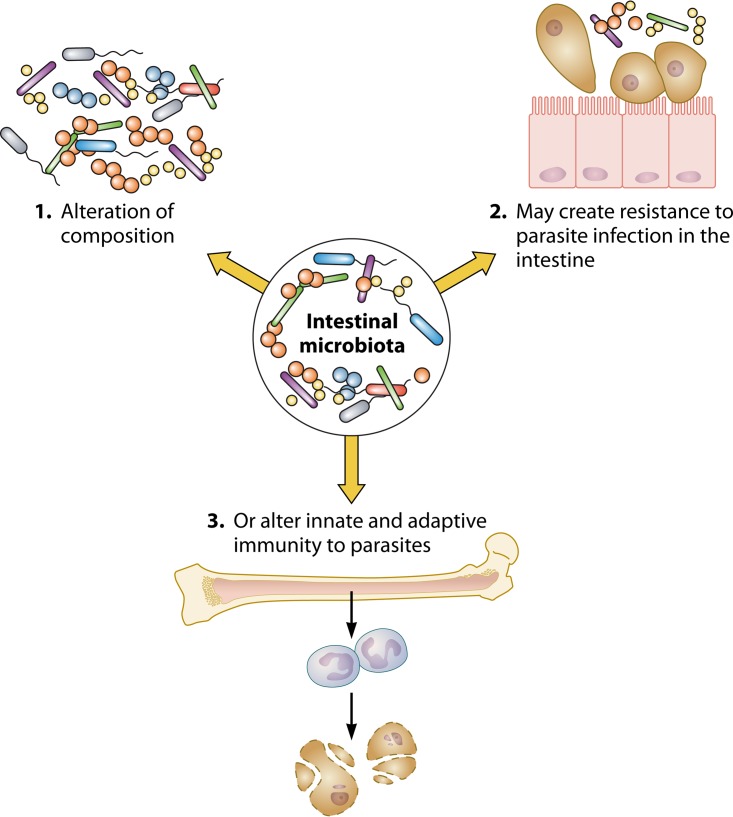
Host intestinal microbiota and interactions with host and parasite. Changes in the composition of the intestinal microbiota (image 1) may increase resistance to parasite infection at mucosal sites, such as the intestine, by mechanisms such as decreased virulence or parasite adherence (image 2). Changes in the microbiota may also alter systemic immunity to parasites by alteration of granulopoiesis or adaptive immunity (image 3). A better understanding of the mechanisms underlying microbiota-mediated protection may help explain clinical variability and help treat parasitic protozoan infections.

Unlike for major bacterial and viral pathogens, established and readily available vaccines do not exist to prevent parasitic protozoan infections. A better understanding of the factors that influence immunity to these diseases may provide a foundation to design novel public health interventions. Transmission of the enteric protozoa typically occurs through the fecal-oral route. The intestine is densely populated by commensal bacteria well situated to influence the behavior of the protozoan parasites with which they directly interact (1). The potential influence of the microbiota on parasites is not, however, limited to the intestinal protozoa. Protozoa that live in the blood or tissue of humans may also be affected by the interplay between the gut microflora and the host metabolism and immune system (1–6). The focus of this review will therefore be the impact of the human microbiota on the parasitic protozoa that infect the intestine (Entamoeba histolytica, Giardia, Cryptosporidium, Blastocystis hominis) or vagina (Trichomonas vaginalis) or cause systemic infections (Plasmodium falciparum) (7). Changes in the composition of the intestinal microbiota may increase resistance to infection at mucosal sites, as well as alter systemic immunity to these parasites (Fig. 1).

## PARASITIC PROTOZOANS AND THE SCOPE OF THE PUBLIC HEALTH IMPACT

Worldwide, diarrhea is currently the second leading cause of death in children younger than 5 years of age and is associated with around 500,000 deaths per year (8–10). Although diarrhea can be caused by many pathogens, in a large proportion of cases, the causal organism is a parasitic protozoan (11). In 2010, an estimated 357 million cases of illness with at least one of three enteric protozoa, Entamoeba, Cryptosporidium, and Giardia, resulted in 33,900 deaths and the loss of 2.94 million disability-adjusted life years (12). In a recent study of moderate-to-severe diarrhea in African and Asian children, Cryptosporidium spp. were some of the top diarrhea-associated pathogens (13).

Despite the significant health burden that protozoans cause, infections can be asymptomatic. For instance, in a Bangladeshi childhood cohort, Entamoeba histolytica, the causative agent of amebiasis, was found to be associated with diarrhea in only 1 of 4 infections (14, 15). Cryptosporidium and Giardia infections are also marked by wide variations in clinical presentation (16–19). Plasmodium infections result in clinical presentations that range from asymptomatic to severe malaria and result in ∼1 million deaths annually. Despite this toll, the factors that determine disease severity remain poorly understood (20). Host genetics and variation in immune response contribute to protection from parasites; however, it is increasingly clear that the intestinal microbiota may have a significant influence on the disease progression of both the enteric protozoa (1) and blood-borne malaria parasites (4).

## INTESTINAL MICROBIOTA

The intestinal bacterial microbiota (21, 22) is a complex community of bacteria which is comprised of at least several hundred species. These organisms form a symbiotic relationship that influences human physiology and disease progression (23, 24). Epidemiological studies have shown that the composition of the intestinal bacterial microbiota can correlate with the development of, or resistance to, obesity (25), malnutrition (26, 27), and allergic disease (28) and may also influence cognitive function and development (29). The intestinal microbiota is not limited to prokaryotes (30), with archaea and eukaryotes potentially contributing to clinical variation (31, 32).

Microbiota compositions can vary significantly from one person to the next (33), even within healthy individuals or twins in the same household (34). Several studies have noted that the bacterial microbiota may influence the virulence of individual pathogens and potentially add variability to the outcomes of parasitic protozoan infections (1, 22). For example, coculture with Escherichia coli strains can augment or attenuate the virulence of Entamoeba histolytica (35, 36). Recently reported studies highlight the impact of the microbiota on infections with enteric protozoa and on infection with extraintestinal Plasmodium parasites.

## MUCOSAL PARASITES AND MICROBIOTA INTERACTIONS IN HUMAN POPULATIONS

Mucosal infection with the enteric protozoa Entamoeba, Giardia, Cryptosporidium, and Blastocystis can be asymptomatic or cause diarrhea, abdominal pain, and/or weight loss. The infecting parasites reside in the intestinal mucosa and therefore are surrounded by the mucosa-associated microbiota. It has been proposed that the dynamic interplay that occurs between the protozoan parasite, host microbiota, and host immune system shapes the clinical outcome of enteric infections (1, 37).

Infection with the gut parasite Entamoeba was significantly correlated with fecal microbiome composition and diversity. Entamoeba species infection was predicted by the composition of an individual's gut microbiota with 79% accuracy in a study of the farming and fishing populations in southwest Cameroon (38). One of the most important taxa in predicting an infection with Entamoeba was Prevotellaceae. In a separate independent study focused on the E. histolytica-associated diarrhea that is common in Bangladeshi infants, levels of Prevotella copri, a member of the Prevotellaceae, were found to be elevated in patients with diarrheagenic E. histolytica infections (39) (Table 1). The Cameroonian study was focused on infected adults who were not experiencing symptomatic amebiasis; therefore, it is interesting that both P. copri and Prevotella stercorea were significantly downregulated in infected individuals (38, 40, 41). Both studies suggest that microbiota composition may play a significant role during an E. histolytica infection. These studies also highlight the potential influence of inflammation driven by the gut microbiome in altering parasite infection outcomes (37, 39). Elevated levels of P. copri have been associated with severe inflammation and an increased risk of autoimmune disease and colitis, suggesting that the organism is proinflammatory (41).

**TABLE 1:** Specific components of the microbiota during human protozoan infection

Protozoan	Microbiota component	Influence	Reference
E. histolytica	Prevotellaceae	Predicted infection	38
E. histolytica	Prevotella copri	Predicted diarrhea	39
Cryptosporidium	Proteobacteria, Firmicutes, Escherichia coli CFT073, Bacillus spp., Clostridium spp.	Increased relative abundance in Cryptosporidium-negative subjects	42
G. duodenalis	Bifidobacterium	Increased relative abundance in Giardia-positive subjects	47
Blastocystis	Clostridia, Enterobacteriaceae	Increased Clostridia levels but lower Enterobacteriaceae levels in Blastocystis-positive subjects	50
T. vaginalis	Lactobacilli, Mycoplasma, Parvimonas, Sneathia	Decreased lactobacilli and increased Mycoplasma, Parvimonas, and Sneathia abundances in T. vaginalis-positive subjects	52
Plasmodium falciparum	Bifidobacterium, Streptococcus	Higher proportion of Bifidobacterium and Streptococcus organisms in a low-infection-risk group	4

Cryptosporidium, Giardia, Blastocystis, and Trichomonas infections may also be influenced by the gut microbiota. A retrospective study of volunteers who were originally enrolled in Cryptosporidium infectivity studies (42) examined the relationship between the relative abundances of several bacterial taxa commonly found in adults prior to or within 48 h of infection and infection outcomes. The patients that were protected from infection had a greater abundance of Proteobacteria and lower Bacteriodetes and Verrucomicrobia levels than infected subjects. There was a higher ratio of Firmicutes to Bacteriodetes in uninfected subjects than in infected subjects. Seven specific taxa had differences of at least 2.5-fold between the two groups. Specifically, uninfected subjects had increased relative abundances of the indole-producing bacteria Escherichia coli CFT073 and Bacillus spp., as well as Clostridium spp. In contrast, infected subjects had increased relative abundances of Bacteroides fragilis, Bacteroides pyogenes, and Prevotella bryantii, as well as Akkermansia muciniphila (Table 1). Presently, the mechanism by which increased indole production may protect from Cryptosporidium is unknown. Indole may directly adversely affect the parasite or perhaps alter host tissues to enhance the innate response by increasing epithelial integrity (43) and/or stimulating anti-inflammatory pathways (42, 44).

A study of intestinal parasite infection in individuals in southern Côte d'Ivoire utilizing PCR-temporal temperature gel electrophoresis (TTGE) and quantitative PCR demonstrated that TTGE profiles clustered into four significantly different groups, i.e., groups that are positive for Giardia duodenalis, positive for Entamoeba spp. and Blastocystis hominis, negative for protozoa, and positive for all three parasites. Quantitative PCR of selected bacterial species in these four groups showed that there was a significant increase in the relative abundance of Bifidobacterium in G. duodenalis-positive patients. This study suggested that the tested intestinal protozoans can induce significant changes in the microbiome which result in substantially different bacterial communities (Table 1).

The relative abundances of Faecalibacterium prausnitzii and E. coli have been used as a marker of the inflammatory bowel disease (IBD)-induced dysbiosis associated with increased E. coli levels (45, 46). Application of this tool to samples from a patient cohort in Côte d'Ivoire suggested that the Côte d'Ivoire and Cameroonian study results were in agreement and that an increase in microbiome diversity occurs in asymptomatic Entamoeba species infections. The Côte d'Ivoire results also suggest that this observation may be extended and that an increase in microbiome diversity also occurred during Blastocystis hominis infections (47). It is controversial, however, whether Blastocystis can cause diarrhea (48). Part of the reason for this controversy might be due to the tremendous genetic diversity within Blastocystis spp. Blastocystis hominis consists of at least seven morphologically identical but genetically distinct organisms (49). The gut microbiome which Blastocystis encounters upon infecting a human host may also influence clinical outcomes. Audebert et al. compared the microbiomes of Blastocystis-colonized and Blastocystis-free patients in a case-control study design that controlled for environmental and clinical risk factors, such as seasonal variation (50). The authors also reported a higher bacterial diversity in the fecal microbiota of Blastocystis-colonized patients, with a higher abundance of Clostridia as well as a lower abundance of Enterobacteriaceae (Table 1). These results suggested that Blastocystis colonization may be associated with expansion of members of the intestinal microbiota generally associated with a healthy gut microbiota, rather than with expansion of bacteria associated with gut dysbiosis.

Trichomonas vaginalis, the causative agent of trichomoniasis and an extracellular parasite of the human urogenital tract, is the most common nonviral sexually transmitted infection globally (51). Women are disproportionally impacted by trichomoniasis, with symptomatic infection primarily impacting the vaginal mucosa. Variation in clinical presentation of disease may be impacted by the composition of the vaginal microbiota. In a study of the vaginal microbiota of T. vaginalis-positive and T. vaginalis-negative women, infection was associated with vaginal microbiota consisting of low proportions of lactobacilli (52) (Table 1). T. vaginalis interactions with various Lactobacillus species inhibit parasite interactions with human cells (53).

In summary, the referenced human studies suggest that there is a strong link between the composition of the intestinal bacterial microbiota and mucosa-associated enteric protozoa (Table 1). Future studies are needed to understand the nature of the connection and how it can be utilized for disease prevention.

## PLASMODIUM AND GUT MICROBIOTA

Approximately 60% of the world's population is at risk of infection with Plasmodium (54, 55). However, the distribution of clinical malaria is highly heterogeneous. In studies in Kenya and Senegal, the number of clinical episodes of disease ranged from 0 to 40 per child over a 5-year period in the same community (56, 57). Clinical variation has been attributed to genetic differences. For example, heterozygous carriers of the hemoglobin variant HbS, associated with sickle cell disease, are healthy and are protected from severe forms of malaria, including cerebral malaria (58). Variation in exposure and variance in immune response are also implicated. However, these factors may not completely explain such a large clinical variation (55, 59). The intestinal bacterial microbiota might represent an environmental factor that may contribute to this variability.

In a recent study, stool samples were collected from a cohort of Malian children and adults just before the P. falciparum transmission season (4). The compositions of gut bacterial communities in these individuals were determined and compared to the risks of acquiring P. falciparum infection and febrile malaria. A significant association was found between microbiota composition and the prospective risk of P. falciparum infection. The intestinal microbiota of subjects who did not become infected had a significantly higher proportion of Bifidobacterium and Streptococcus species than subjects who became infected with P. falciparum. However, no relationship was observed between microbiota composition and the risk of developing febrile malaria once P. falciparum infection was established. The authors note that this is possibly due to a lack of statistical power. The preliminary finding of an association between gut microbiota composition and P. falciparum infection risk suggests that alteration of the composition of the intestinal microbiota may decrease the risk of P. falciparum infection in areas where malaria is endemic and may potentially augment partially effective malaria vaccines (4) (Table 1).

Gut bacteria might influence extraintestinal disease via many pathways, such as by alteration of adaptive immunity and augmentation of the magnitudes of T cells and B cell-mediated responses and perhaps by enhancement of innate immune pathways via trained immunity (60). Mechanisms underlying these extraintestinal effects are poorly understood. Metabolic products, such as short-chain fatty acids (61, 62), or host-derived factors, such as damage-associated molecular pattern molecules induced by the microbiota (63, 64), might be partially responsible for these effects. The metabolite pools present in animal models with differential, microbiota-dependent susceptibility to Plasmodium infection varied significantly in one study, with decreases in nucleotides, amino acids, and the substrates involved in the biosynthesis of these compounds in resistant mice, along with more-robust T and B cell responses (20, 65). The gut microbiota has also been shown to have a systemic influence on serum metabolites in both animal models and humans (66, 67). Blood-stage parasites have been shown to be highly susceptible to metabolic dysregulation induced by antimalarials (68) and might also be influenced by changes induced by the microbiota. Therefore, the intestinal microbiota may influence the clinical outcome of a Plasmodium infection via alteration of the metabolome and modulation of innate or adaptive immunity.

## ALTERATION OF THE MICROBIOTA AS A THERAPY FOR PROTOZOAN INFECTIONS?

Patient cohorts and future microbiome epidemiological studies will establish a more complete understanding of variation in clinical presentations of infection with parasitic protozoa. However, population-based studies do not allow us to test the effects of the microbiota on parasite survival and proliferation. Therefore, *in vitro* and *in vivo* disease models provide a useful tool to understand how the intestinal bacterial microbiota may influence severity and progression of infection and what mechanisms might underlie that progression.

*In vitro* culture models allow interactions between infecting agents and individual components of the microbiota to be analyzed. A study of the *in vitro* effects of six Lactobacillus acidophilus strains and Lactobacillus johnsonii La1 on Giardia duodenalis survival, for example, demonstrated that L. johnsonii La1 significantly inhibited the proliferation of Giardia trophozoites. The potential protective role of L. johnsonii La1 (NCC533) was independently confirmed by *in vivo* experiments with La1-treated gerbils, which were protected against Giardia infection and mucosal damage (69–71). In another *in vitro* study, common human commensal bacteria were cocultured with E. histolytica. Culture of Lactobacillus casei and Enterococcus faecium alone with amebas reduced parasite survival by 71%. When both bacteria were used in combination, survival was reduced by 80%. A previous study demonstrated a link between decreased Lactobacillus and amebiasis in Indian patients (72), further supporting a potential link between these bacteria and resistance to ameba infection.

As mentioned previously, lactobacilli may impact susceptibility to T. vaginalis infection in women (52). Mechanisms underlying this effect are still being studied; however, inhibition of adhesion of the parasite might help explain protection. In one study, adhesion assays were carried out by incubating vaginal epithelial cells (VECs) with T. vaginalis and lactobacilli together and by comparing levels of parasite adhesion to nonlactobacillus recipient controls. Lactobacillus gasseri ATCC 9857 and CBI3 caused significant parasite adhesion inhibition in a dose-dependent manner (53).

Studies such as these may lay the foundation for utilizing individual components of the microbiota to provide cost-effective prophylactic treatment for parasite infection without the overuse of antimicrobial agents (Table 1) (73). Unfortunately, current coculture experiments do not allow us to explore the influence of the host immune system. Although differences exist between the murine and human gut microbiotas, murine models provide a powerful tool to explore host-microbiota-pathogen interactions in the context of an active immune system (74).

## HOST-MICROBIOME INTERACTIONS AND MUCOSAL PARASITE INFECTION IN MURINE MODELS

The development of murine models of parasitic protozoan infections has allowed for more-detailed immunophenotyping of the mammalian host response to changes in the microbiota and its influence on infectious disease (1, 75). Murine models have also offered unexpected advancements in our understanding of interactions between the microbiota and the host due to variation in the communities of bacteria present in commercial animal vendors facilities, notably segmented filamentous bacteria (SFB) (44, 45, 93). Alteration of the intestinal microbiome in model systems and careful observation of variation between models in different environments therefore allow for a better understanding of immune factors that may help explain clinical variation in parasitic disease.

Variation in the microbiota in commercial animal facilities can result in significant changes in the progression of inflammatory and infectious diseases (76, 77). A salient example of this is colonization with a single murine commensal Clostridium SFB (78, 79). It was observed that C57BL/6 mice from Jackson Laboratories did not have significant interleukin 17A (IL-17A) induction in their intestinal mucosa but that C57BL/6 mice from Taconic Farms did (80). This suggested that a difference in the microbiotas of mice between these two vendors might underlie the difference in cytokine induction. Ivanov et al. (80), utilizing specific-pathogen-free mice from both vendors and germfree mice, showed that SFB, which were present in mice from Taconic Farms, were the component of the microbiota underlying the changes in immune function. Research in murine models has also shown that the immune response induced by SFB alters the severity of extraintestinal autoimmune encephalomyelitis (77, 80–82).

**FIG 2:**
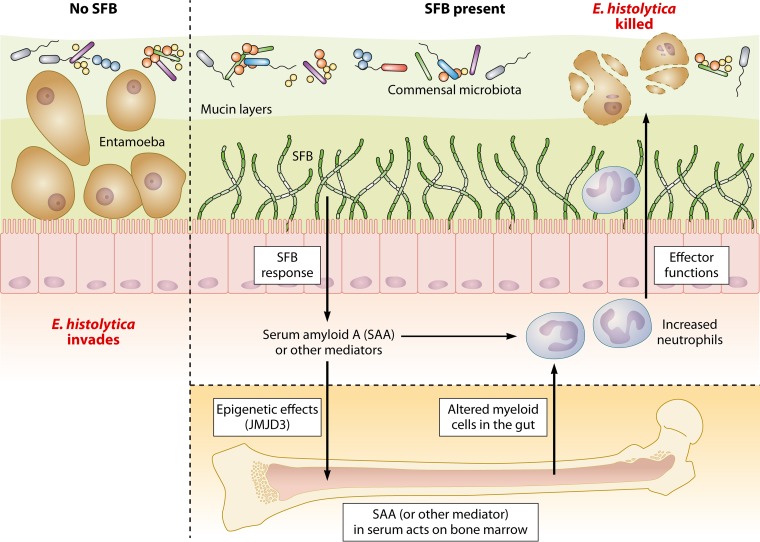
Model of SFB-mediated protection against E. histolytica colonization. SFB (segmented filamentous bacteria) colonization of the intestine may induce soluble mediators, including SAA, which may increase intestinal immune responses against ameba as well as trigger systemic epigenetic changes in bone marrow that support more-robust granulopoiesis and protection against intestinal E. histolytica infection. (Republished with modifications from *mBio* [83].)

Recently, with a murine model of E. histolytica infection, we demonstrated that mice colonized with SFB are protected from experimental amebiasis (83). In exploring the responsiveness of immune cells in these mice, it was discovered that bone marrow-derived dendritic cells (BMDCs) from SFB-colonized mice produced significantly higher levels of IL- 23. There was also an increase in neutrophils in the intestine, which resulted only after ameba infection (28). IL-23 is a cytokine (29) linked to induction of IL-17A and neutrophils, which in turn have been shown to be important in immunity to the ameba (30, 31). Transfer of BMDCs derived from mice colonized with SFB provided protection from E. histolytica infection. This work suggested that a gut-associated commensal might alter the responsiveness of bone marrow-derived cells to subsequent inflammatory challenges (Fig. 2).

In this model of amebiasis, a host damage-associated molecular pattern molecule, serum amyloid A (SAA) (84) was also increased in the sera of SFB-colonized mice compared to the level in the sera of mice lacking the commensal. Transient gut colonization with SFB or SAA administration alone increased the H3K27 histone demethylase Jmjd3 in the bone marrow and persistently increased bone marrow Csf2ra expression as well as granulocyte monocyte precursors (GMPs), and protected from ameba infection. Protection was associated with increased intestinal neutrophils (63). Pharmacologic inhibition of Jmjd3 H3K27 demethylase activity during SAA treatment or blockade of granulocyte-macrophage colony-stimulating factor (GM-CSF) signaling in SFB-colonized mice prevented GMP expansion, decreased gut neutrophils, and blocked protection from ameba infection. These results indicate that alteration of the microbiota and systemic exposure to host SAA can influence granulopoiesis and susceptibility to amebiasis, potentially via epigenetic mechanisms. Gut microbiota-marrow communication is a previously unrecognized mechanism of innate protection from ameba infection (63, 83) (Fig. 1 and 2). The intestinal microbiota likely has significant extraintestinal effects on the host immune response to parasites. These changes may be relatively long term, perhaps via induction of immune memory pathways, such as trained innate immunity (60), or via influences on adaptive immunity that are yet to be fully understood.

Antibiotic treatment which disrupts the commensal microbiota is often utilized to establish infection with pathogens in model systems. Observation of differences between the immune response in antibiotic-treated mice and untreated mice may therefore lead to insights into the role of the microbiota in the host response. In a model of Giardia duodenalis infection, for example, antibiotic alteration of the microbiome was shown to prevent CD8 T cell activation by Giardia (6). Giardia-infected mice that were not treated with antibiotics had more activated CD8^+^ αβ T cells in the small intestinal lamina propria than uninfected mice. The increase in CD8^+^ T cells was absent in antibiotic-pretreated, Giardia-infected mice. One potential mechanism is that during infection, the parasite promotes breakdown of the intestinal barrier. Translocation of luminal bacteria into the mucosa leads to activation of CD8^+^ T cells; therefore, reducing the bacterial load by antibiotic treatment may reduce this and prevent pathological CD8^+^ T cell activation (6).

Giardia duodenalis infections can have a long-term impact on human heath, and the reduction of host disaccharidases associated with Giardia infections may play an important role. Disaccharidases are required for the complete assimilation of nearly all carbohydrates present in food and drinks. The deficiency in disaccharidases has been thought to result from epithelial damage and shortening of the intestinal epithelial microvilli. However, in Giardia-infected mice, deficits in disaccharidase can be reversed by blocking CD8^+^ T cell activation by either CD4 signaling or antibiotic treatment (6). This study suggests that differences in antibiotic usage and their effects on the human microbiome might be important factors to consider when evaluating the clinical outcome of a Giardia infection.

## MURINE PLASMODIUM INFECTION AND THE MICROBIOTA

Recently, the influence of the microbiota on Plasmodium infection was explored by utilizing genetically similar inbred strains of mice (C57BL/6) maintained by different vendors, Jackson Laboratory, Taconic Farms, the National Cancer Institute/Charles River (NCI), and Harlan (20). C57BL/6 mice from each of these vendors were infected with Plasmodium yoelii. Following infection, significant differences in parasitemia were observed between the genetically identical mice from different vendors, with mice from Jackson Laboratory and Taconic Farms being resistant to the parasite. Germfree mice that received cecal transplants from “resistant” or “susceptible” mice had low and high parasite burdens, respectively, demonstrating that the intestinal microbiota may shape the severity of malaria. Resistant mice exhibited increased abundances of Lactobacillus and Bifidobacterium compared to those in susceptible mice. Additionally, susceptible mice treated with antibiotics followed by probiotics made from these bacterial genera displayed a decreased parasite burden. Consistently with differences in parasite burden, resistant mice exhibited an increased antibody profile and increased CD4^+^ T cells and B cells compared with those of susceptible mice. Therefore, the composition of the gut microbiota may be an unidentified risk factor for severe malaria and alteration of the intestinal microbiota might augment the host response to extraintestinal parasites.

## ROLE OF THE PROTOZOAN MICROBIOTA IN INFECTION AND INFLAMMATION

The primary focus of this review has been parasitic protozoa and influences of the bacterial microbiota on host immunity to these protozoa. However, it is important to note that an emerging body of work suggests that protozoa may also alter host immunity to subsequent exposures (30). Fecal-oral ingestion of Giardia cysts leads to varied clinical syndromes ranging from acute or chronic diarrhea to long-term asymptomatic colonization (16). A recent study of children in Bangladesh showed that early-life Giardia exposure neither increased nor decreased the odds of acute diarrhea from any cause. However, Giardia infection was a risk factor for stunting but not poor weight gain (85). It has also been noted that patients that have been infected with Giardia often have gut dysfunction well after their infection is cleared (86, 87). Giardia infection has been associated with protection from diarrhea in other cases (17, 88, 89). Mechanisms underlying these disparate outcomes in Giardia infection in humans are not presently well understood. However, recent work in murine models provides a demonstration of how protozoan infection might provide protection from infection while exacerbating colitis.

Tritrichomonas musculis is a common murine commensal found in wild mice and some animal colonies. It has recently been shown to cause expansion of tuft cells, a unique epithelial cell subtype important in the generation of type 2 immune responses (90). This work suggests that commensal protozoa may be important in establishing the basic structure of the mammalian intestine. The protozoon has also just been shown to lead to expansion of adaptive Th1 cells and Th17 effector cells in the colonic mucosa. This expansion was dependent on distinct, migratory DC subsets but also required the production of IL-18 by epithelial cells. These results together with the high expression of the IL-18 receptor IL-18Rα on colonic-infiltrating effector T cells suggested that T. musculis-specific T cell immunity is likely initiated in the draining lymph nodes by migratory colonic DCs and is likely propagated at the tissue site by epithelial IL-18 (91). Interestingly, T. musculis colonization also conferred significant protection from Salmonella infection-driven enteritis in an IL-18-dependent manner (91). However, colonization with T. musculis, along with having a role as a “protistic” antibiotic, exacerbated the development of T-cell-driven colitis and resulted in the development of sporadic colorectal tumors in colonized mice. This effect of T. musculis was also observed in an independent study of murine colitis (92). Combined, these studies revealed novel host-protozoan interactions that led to increased mucosal host defenses while also increasing the risk of inflammatory disease.

## CONCLUSIONS

Recent studies have highlighted the potential contribution of the intestinal microbiome to clinical variation in parasitic protozoan infections. The microbiome and parasites may interact in various ways, which may include (i) alteration of parasite virulence, (ii) induction of dysbiosis or perhaps even beneficial shifts in the microbiota that increase competition for the niche of the lumen of the gut, and finally, (iii) modulation of host immunity to the parasite. The courses of both mucosal and systemic parasite infection may also be shaped by specific members of the microbiota, and in turn parasite infection may alter the microbiota in such a way that the unique signature can be diagnostic of the presence of the parasite.

The exact mechanisms underlying microbiota modulation of host immunity are not yet fully understood; however, it is becoming increasingly apparent that components of the microbiota can alter both innate and adaptive immune cell populations so that a more robust response is mounted following subsequent challenge with infectious agents, including parasitic protozoa. Mechanisms underlying this shift might include the recently described concept of trained innate immunity, in which epigenetic changes enable innate immune cells to more effectively clear unrelated pathogens, and by enhancement of adaptive immunity. Ultimately, further exploration of interactions between the gut microbiome and parasitic protozoans will provide additional tools and approaches that will help in the diagnosis and treatment of infectious and inflammatory diseases. Study of protozoan interactions with the host immune system and the microbiota also help us to better understand fundamental mechanisms of mammalian immunology.
